# ***I***dentification of biop***S***ycho***S***ocial factors predictive of post-tra***U***matic stress disorder in patients admitted to the ***E***mergency department after a trauma (ISSUE): protocol for a multicenter prospective study

**DOI:** 10.1186/s12888-019-2154-z

**Published:** 2019-05-30

**Authors:** Mohammad-Hashim Wafa, Marie Viprey, Laurent Magaud, Julie Haesebaert, Edouard Leaune, Emmanuel Poulet, Clemence Bied, Anne-Marie Schott

**Affiliations:** 1 HESPER EA 7425, Univ Lyon, Université Claude Bernard Lyon 1, Lyon, France; 2 Pôle de santé publique, Hospices Civils de Lyon, Lyon, France; 3 PsyR2 Team, U 1028, INSERM and UMR 5292, CNRS, Center for Neuroscience Research of Lyon (CRNL), CH Le Vinatier, Lyon-1 University, Bron, France; 4 Department of Psychiatry Emergencies, CHU Lyon, Hôpital Edouard Herriot, Lyon, France; 5 SHU, CH Le Vinatier, Lyon 1 Université, Bron, France

**Keywords:** Post-traumatic stress, Biopsychosocial, Emergency, Trauma, Addiction, Anxiety, Depression, Dissociation

## Abstract

**Background:**

Traumatic exposure is a frequent issue in patients visiting emergency departments (EDs). Some patients will subsequently develop post-traumatic stress disorder (PTSD) while other will not. The problem is under-diagnosed in EDs and no standardized management is provided to prevent PTSD. Most studies focused on a particular group of trauma whereas we need a global approach to further develop interventions for detecting and treating patients at high risk. We aim to assess the prevalence of traumatic exposure and situation at high risk of further PTSD and identify pre and peri-traumatic biopsychosocial factors predisposing individuals to PTSD in the general context of EDs.

**Methods:**

This comprehensive multicenter study will have two steps. The first step will be a cross-sectional study on moderate and high risk of PTSD prevalence among EDs visitors with a recent history of trauma. All patients aged 18–70 years, presenting with a recent history of trauma (< 1 month) in one of the six EDs in the Auvergne-Rhône-Alpes region (≈1/10° of the French population) will be included over a 1-month period and approximately 1500 subjects are expected in this cross-sectional step. The risk of PTSD will be assessed using the Impact of Event Scale Revised (IES-R). Self-administered questionnaires will be used to measure acute stress (IES–R), and a number of potential bio-psycho-social risk factors. Demographic and physical health-related data will be collected from medical file. Second step will be a prospective cohort study within a sub-sample of 400 patients enrolled in step 1, randomly selected with stratification on sex, age, ED, and IES–R score. At 3 months, PTSD will be defined by a ≥ 33 score at PTSD Check List for DSM–5 (PCL–5) through a telephone interview. We will evaluate definite PTSD biopsychosocial predictive factors using a multivariate logistic regression model and describe evolution of PTSD at 3 months.

**Discussion:**

This is the first study to assess PTSD predictors prospectively with a biopsychosocial approach within a cohort representative of EDs visitors. The results will inform the development of dedicated interventions to decrease the risk of subsequent PTSD.

**Trial registration:**

ClinicalTrials.gov: NCT03615014; ISSUE protocol 2nd version was approved on 07/08/2018.

## Background

Post-traumatic stress disorder (PTSD), one of the most serious sequelae of a traumatic exposure, is a chronic disorder with a high level of anxiety and neurovegetative symptoms that interrupt normal psychosocial functioning of the person [1–4]. There are four main categories of diagnostic symptoms, namely, symptoms of re-experiencing the trauma, avoidance and numbing symptoms, negative alterations in mood and cognition, and hyper-arousal symptoms. [4–9]. The mean duration of PTSD is 5.3 years (range: 0.2–28.1) [10]. Patients with PTSD are more likely to develop other psychiatric disorders such as depression, anxiety disorders, substance use disorders, and/or attempt suicide [11, 12]. The likelihood of developing somatic pathologies such as cardio-vascular disorders is also very high [13, 14]. Therefore, such patients, in addition to the disorder itself, suffer from its physical, occupational, and social sequelae. Such consequences result in a significant economic impact [5].

Worldwide approximately 60.7% of all men and 51.2% of all women encounter at least one traumatic event in their lifetime. However, not all of them will develop PTSD; it is estimated that after a trauma, 8% of men and 20% of women will subsequently develop PTSD [15]. Prevalence of the condition is highly variable (4–86%), but is higher among those who experienced the stressors directly, such as victims of intimate partner violence (IPV), sexual victimization, servicemen, refugees, and asylum seekers [5]. In the French population, the lifetime exposure to a traumatic event is estimated to be 72.7% and lifetime prevalence of PTSD to 3.9% [10], which is lower than that found in the United States (7.8%), but higher than rates in Spain (2.2%) or Italy (2.4%) [10].

Among the patients consulting EDs after a recent trauma, 18 to 42% suffer from acute stress disorder (ASD) [16–18], which is highly predictive of subsequent occurrence of PTSD [16, 19, 20]. However, ASD is often underdiagnosed in ED, mainly due to the assessment focused on urgent physical problems, complaints of the patient (pain, insomnia), and overlooking the traumatic context [1, 21].

PTSD predictive factors are worthwhile for identifying populations at high risk, which in turn could lead to early diagnosis and management of these cases, and therefore could help reduce the occurrence of the disorder. Screening for such factors, however, is not incorporated into any structured assessment procedure in EDs.

Previous research has identified the following predictive factors for PTSD: pre-traumatic factors (e.g. female sex, extreme age, low Intelligence quotient (IQ), childhood or prior traumatic exposure, pre-existing mental health problem, substance abuse, anxious personality), specific features of the index trauma (perception of death threat, head trauma, intentional aggression), and post-traumatic psychosocial factors such as peri-traumatic dissociation, acute stress disorder and low social support [1–3, 5, 15, 21–40]. However, these studies have methodological limitations. For instance, they suffer from selection bias as they usually focus only on a particular population [32, 41–44] or on a single trauma type such as road traffic accident [32, 43–46]. Most used case-control or retrospective designs that suffer from information/recall bias [41, 42, 44, 46–49], and were conducted on small samples and/or had high loss-to-follow-up rates for prospective studies reducing the generalization of the results [20, 26, 28, 43, 45, 46, 50–54]. Furthermore, studies usually focused on either biological, psychological, or social factors; none considered a comprehensive biopsychosocial approach to study the predictive factors [19, 20, 25, 32, 48, 50, 52, 54, 55]. It is also of note that, to the best of our knowledge, there is no published prospective epidemiological study that has evaluated the prevalence of acute stress in survivors of diverse trauma visiting an ED.

We therefore aim to address all these limitations in a prospective multicenter study that will recruit a large number of patients in the ED who were exposed to various types of trauma. We will measure prevalence of acute stress and level of PTSD risk through an initial cross-sectional study. We will then adopt a holistic viewpoint to determine the predictive factors such as specific features of the trauma as well as demographic, biological, psychological and social risk factors through a cohort study.

## Objectives

### Primary objectives

The primary objective of the cross-sectional study is to estimate the prevalence of patients with high or moderate risk of developing PTSD in all consecutive cases admitted to the EDs after recent trauma (< 1 month).

The primary objective of the prospective cohort study is to determine predictors of PTSD occurrence at 3 months in a randomly selected sub-sample of patients included in the cross-sectional study and identified as “at moderate or high risk” for developing PTSD at admission to the ED.

### Secondary objectives

The secondary objectives are to measure acute stress, anxiety disorder, and dissociative experiences in patients at inclusion. At 3 months, the incidence of PTSD, its complications and comorbidities will be estimated, as well as the impact of trauma on occupational and psychosocial functioning of the study subjects.

## Methods/design

This multicenter study will be conducted in two stages. The first stage will consist of a cross-sectional study within all consecutive patients admitted to the participating EDs following a recent trauma (< 1 month), to systematically measure their risk of PTSD. The second stage will be a prospective cohort study designed to analyze the relationship between PTSD occurrence and its putative predictive factors in a sub-sample of patients randomly selected among those identified as “at moderate or high risk” for developing PTSD at admission to the ED and followed-up for 3 months.

### Study setting

The study will take place in six large EDs of the Auvergne-Rhône-Alpes region of France; the four EDs in Lyon (two at the Edouard Herriot hospital, one in Lyon Sud hospital, and one in Saint Luc Saint Joseph hospital), one in Saint Etienne (North university hospital) and one in Clermont Ferrand (university hospital). The region had 7.878 million inhabitants in 2015 (source Eurostat) and covers urban and rural, economically deprived and non-deprived areas.

### Participant eligibility

The target population will be adults (≤ 70 years of age) visiting the EDs during the 1-month inclusion period who were victims of a recent traumatic event (< 1 month) and willing to participate in the study. The trauma will be defined as a direct exposure, directly witnessing trauma to a third party, or discovering that a traumatic event has happened to a close family member or a close friend. In case of actual death or death threat to a member of the family or a friend, the event(s) must have been violent or accidental. We will also consider the recurrent or extreme occupational exposure to traumatic events (e.g. front-line workers collecting human remains, police repeatedly exposed to explicit child sexual abuse) [4]. Furthermore, participants must be affiliated to the French public health insurance system, and provide written informed consent. In case of an adult under curatorship, the recruiter will seek only his/her consent, the consent of the curator being not mandatory in the French law.

Patients who are either unable to communicate fluently in French or under guardianship, and/or have clinical instability that makes completing the questionnaire(s) impossible (e.g. agitation, critical condition, distorted consciousness, etc.) will not be included in the study.

### Recruitment process

**Fig. 1:**
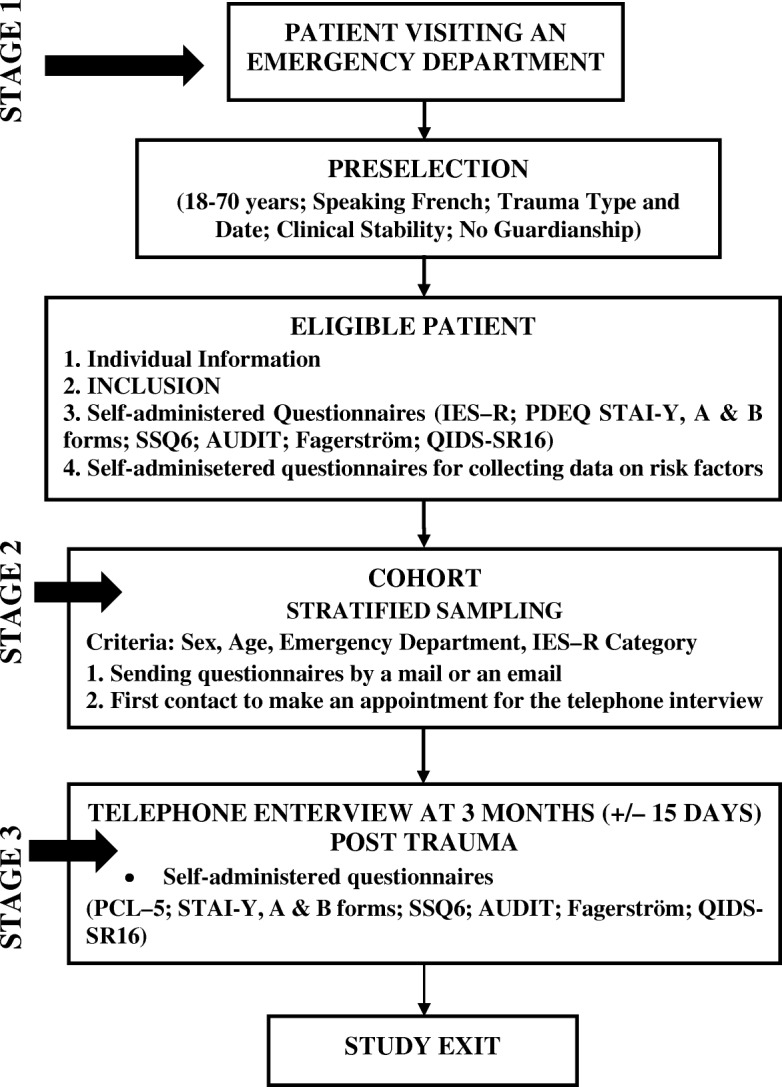
Inclusion stages and follow-up of subjects

Figure 1 illustrates stages of the study. Initially (for a period of 1 month) each eligible consumer of the assigned EDs will be screened from 08:00 to 24:00/day and 7 days/week, based on inclusion criteria. The screening will be performed by a trained interviewer (research assistant or medical/nurse student), supervised by a psychiatrist or emergency physician. The investigating physician will explain the study to each eligible patient and provide him/her with a written synopsis of the objectives and course of the research (including that they can be drawn at random to receive the questionnaires by an email or postal mail and a telephone follow-up). In case the patient is willing to participate, he/she will date and sign the consent form and a trained interviewer will collect baseline data. Participation in the study will neither change any healthcare required by the patients, nor their right to retract from the study at any time they desire.

### Constitution of the cohort

The prospective cohort study will be conducted on a sub-sample of the participants (Fig. 1). The study biostatistician will arrange a cohort of 400 subjects selected through random sampling stratified by sex (male/female), age (determined by interquartile range of the collected data), investigating ED (1 to 6), and IES-R score (< 12, 12 to 34, > 34). Selected subjects will receive the questionnaires by email or postal mail and will be interviewed by telephone at 3 months (± 15 days) after the index trauma. Mental health professionals (a psychologist, psychiatrist, or resident in psychiatry) who have had special training on the PCL–5 application will conduct the interview.

### Data collection

Table 1 summarizes the different stages of data collection.

**Table 1:** Patient inclusion, follow-up, and data collection stages of the study

Steps	Preselection	V1 Inclusion	Establishment of the Cohort	V2 Telephone Follow-up End of the Study
Preselection Criteria Verification *(1)*	X			
Information, Consent Collection and Inclusion		X		
Self-administered questionnaires {IES–R; PDEQ; STAI-Y (A & B forms); AUDIT; Fagerström; QIDS-SR16; SSQ6}		X		
Clinical Data Collection & Self-administered questionnaire (risk factors) *(2)*		X		
Stratified sampling (weighted)			X	
Mailing self-administered questionnaires to subjects to prepare for the telephone interview			X	
Telephone Interview: PCL–5; STAI-Y (A & B forms); AUDIT; Fagerström; QIDS-SR16; SSQ6 (+ back to work time)				X
Intercurrent psychological care (consultation, hospitalization, psychotropic)		X		X

(1) Emergency patient with a recent history of trauma (< 1 month), aged 18 to 70 years, speaks French, and has no guardianship

(2) This self-administered questionnaire consists of PTSD risk factors

Following preselection and written informed consent, patients will be identified as study subjects. At the inclusion phase, he/she will receive self-administered questionnaires to measure acute stress (IES–R), dissociative experiences (PDEQ), anxiety disorder (STAI-Y; A & B forms), social support (SSQ6), alcohol and/or tobacco addiction (AUDIT & Fagerström test), depressive symptoms and suicidality (QIDS-SR16), marital stability, family history of mental health problems and/or instability, socio-economic and familial status, history of trauma exposure, and past psychiatric history. Demographic and physical health-related data will be collected from his/her medical file.

At 3 months after the index date, the cohort study participants will receive self-administered questionnaires by an email {a link with access code to an ePRO (electronic patient reported outcomes) for online completion of questionnaires} or by postal mail (attached with a pre-paid return envelope). The online version of the questionnaires will have to be completed at least one day prior to the telephone interview. These questionnaires will help us assess trauma impact on the patient’s occupational and psychosocial functioning {SSQ6 & STAI-Y (A & B forms)} and PTSD complications and comorbidities such as depression & suicidality (QIDS-SR16), and addiction (AUDIT & Fagerström test).

Additionally, through a telephone interview, a mental health professional (psychologist, psychiatrist, or psychiatric intern) will assess PTSD using the PCL–5 questionnaire and determine whether the subject received any therapeutic care in the 3-month period. The estimated duration of this interview is 15 to 30 min.

Inclusion, follow-up and data collection stages are presented in Table 1.

We will apply the following measures in order to limit the number of dropouts:

Selected patients will receive an email or a postal mail reminding them of their participation in the second stage of the study around 1 month prior to the theoretical date of the interview.

Within 15 days of the interview, we will contact the subject via telephone or email in order to set the date and time for the interview.

In case the first attempt to contact the subject is unsuccessful, we plan three more attempts of telephone call or email. If we fail to establish any (telephone/email) contact up to the intended date of the interview, the subject will be considered as lost to follow-up.

Subject participation in the study will end with the completion of this telephone interview.

Patients with an IES-R score of > 34 will be proposed to consult a specialized healthcare professional (psychiatrist or addictionologist) for the diagnosis and treatment (if necessary) of PTSD or its complications (alcoholism and/or substance abuse, suicidality, and depressive symptomatology).

### Outcome criteria and measure instruments

#### Primary outcome criteria

The primary endpoint for the cross-sectional study is the IES–R score of the subjects reflecting their risk of developing PTSD at inclusion in the ED. An IES-R score > 34 will be considered as a high-risk of subsequent PTSD, an IES-R score of 12–34 as a moderate risk, and an IES-R <  12 a low risk [44, 56, 57]. IES-R is a 22-item self-administered questionnaire composed of three subcomponents: intrusion (8 items), hyperarousal (6 items), and avoidance (8 items). Patients evaluate for each item the experience during the last 7 days on a Likert scale 0 = not at all to 5 = extremely. The total score (from 0 to 88) is the sum of all the evaluations. IES-R has good psychometric characteristics [56–62], and is recommended in France for PTSD surveillance [63]. The IES-R is among the most used scales [64], it is validated in French with a mean completion time of 5 to 10 min [65].

The primary endpoint of the prospective cohort study is the presence or absence of 3-month PTSD defined by PCL-5 (PTSD Check List for DSM-5). PCL-5 is a 20-item self-reported measure. Consistent with the Diagnostic and Statistical Manual of Mental Disorders, Fifth Edition (DSM-5), it assesses 20 symptoms of PTSD. The questionnaire uses “0=not at all” to “4 = extremely” ratings to evaluate each symptom. A probable diagnosis of PTSD is made by considering any item with a score of ≥2 as present and then by adhering to DSM-5 instructions that require at least: 1 item B (questions 1–5), 1 item C (questions 6(− 7), 2 items D (questions 8–14), and 2 items E (questions 15–20). The cut-off score for PCL-5 is ≥33 [66, 67]. This tool has a good sensitivity for a provisional diagnosis of PTSD, and has the advantage to have a shorter completion time (about 5 to 10 min) than the CAPS–IV [16, 19, 20].

#### Secondary outcome criteria

Anxiety disorder will be assessed using the State-Trait Anxiety Inventory (STAI-Y; form A & B) [68, 69] at inclusion and at 3-month follow-up. STAI-Y is a self-report tool that assesses momentary as well as habitual anxiety. It includes two scales of 20 items, each rated from 1 = not at all/almost never to 4 = very much so/almost always [68, 69]. The State-Anxiety subscale (STAI-Y A), assesses the intensity of subjective feelings of tension, worry, apprehension, and nervousness at the current moment. The Trait-Anxiety subscale (STAI-Y B), measures frequency of anxiety vulnerability that includes overall degree of security, confidence, and calmness.

The presence or absence of dissociative experiences will be assessed through the validated French version of Peritraumatic Dissociative Experiences Questionnaire (PDEQ) [64, 70] at inclusion. PDEQ is a self-administered questionnaire designed to assess the presence and intensity of peritraumatic dissociative reactions during or immediately following a potentially traumatic event. In accord with the peritraumatic dissociative symptoms, the questionnaire has 10 corresponding items. For each item, the subject selects the answer most adapted to his/her experience from 1 (not at all true) to 5 (extremely true). The final score is the sum of all the selected answers, varying from 10 to 50, 10 being the minimum signifying absence of dissociative experiences and a score greater than 10 indicates that the patient has dissociative experiences.

At inclusion, we will evaluate patient’s social support as a risk factor and at 3-month follow-up as a psychosocial and occupational consequence of the trauma. For this purpose, we will ask his/her return time to the workplace and use the validated French version of the Social Support Questionnaire 6 (SSQ6) [71, 72]. SSQ6 is a 6-item questionnaire that measures two aspects of perceived social support, i.e. availability and satisfaction. Availability is defined as the individual’s estimation of the number of people who can help him/her if required. Satisfaction is defined as the perceived adequacy between the support received and his/her expectations and needs. For each item, the respondent lists the people (max. 9) he/she can count on in the situation described and expresses his/her degree of satisfaction (from 1 to 6) with regard to this support. We then calculate one score for availability (score N, that varies from 0 to 54), and another for satisfaction (score S, that varies from 6 to 36).

For the assessment of alcoholism and nicotine dependence at inclusion and at 3-month follow-up (as PTSD-related complications), we will use Alcohol Use Disorder Identification Test (AUDIT) and Fagerström test, respectively [73, 74].

The AUDIT consists of 10 questions and screens for risky or harmful use of alcohol. It is the reference for detecting alcohol misuse. Men scoring ≥7 and women scoring ≥6 raise the suspicion of alcohol misuse [73, 75].

The Fagerström test is a quick 6-item test that quantifies patient’s level of nicotine dependence [75]. The score ranges from 0 to 10. Dependency is deemed to be null if the score is from 0 to 2, low from 3 to 4, average from 5 to 6, strong from 7 to 8, and very strong from 9 to 10.

In order to assess depression and suicidal ideation (as risk factors at inclusion), and as PTSD-related complications or comorbidities at 3 months, we will use the Quick Inventory of Depressive Symptomatology (Self-Report) (QIDS-SR16). The QIDS-SR16 is a self-administered questionnaire with 16 items describing the 9 symptom domains of DSM-IV associated with depressive feeling [76–78]. The assessment of depression severity is based on the total score as follows: from 1 to 5, absence of depression; from 6 to 10, slight depression; from 11 to 15, moderate depression; from 16 to 20, severe depression; and from 21 to 27, very severe depression. We will also ask for the number of suicide attempts over the last 3 months.

#### PTSD risk factors

Table 2 summarizes the biopsychosocial factors that have the potential to increase PTSD occurrence. These factors will be assessed at inclusion in the ED. Estimated time for documenting all the questionnaires is around 30 min.

**Table 2:** PTSD risk factors evaluated in the study, evaluation instruments and timing

Factor Category	Predictive Factors	Measure	Timeline
Trauma Characteristics	Type and timing of the trauma	Pre-screening questionnaire	1st visit
	After trauma: Hospitalization (±); Intensive care (±)	Consumer file	1st visit
Demographics	Sex	Consumer file	1st visit
	Age	Consumer file	1st visit
	Socio-economic status	Self-administered questionnaire	1st visit
	Educational level	Self-administered questionnaire	1st visit
	Marital status	Self-administered questionnaire	1st visit
Biological	Heart rate	Consumer file	1st visit
	Blood pressure	Consumer file	1st visit
	Blood alcohol level	Consumer file	1st visit
Psychological	Trauma history	Self-administered questionnaire	1st visit
	Chronic anxiety	STAI-Y (A & B forms) [68, 69]	1st & Final visits
	Past and current psychiatric pathology	Self-administered questionnaire	1st visit
	Current psychotropic treatment at inclusion and during the 3-month period	Self-administered questionnaire	1st & Final visits
	Psychological care during the 3 months	Self-administered questionnaire	Final visit
	Dissociative Experiences	Self-administered questionnaire: PDEQ [70]	1st visit
Social	Alcohol misuse	Self-report: AUDIT [73, 75]	1st & Final visits
	Smoking addiction	Self-administered questionnaire: Fagerström test [74]	1st & Final visits
	Family history of psychopathy or instability	Self-administered questionnaire	1st visit
	Marital stability	Self-administered questionnaire	1st visit
	Social support	Self-administered questionnaire: SSQ6 [71, 72]	1st & Final visits
Others	Somatic pathology	Patient file	1st visit
	Emergency care time	Patient file	1st visit

### Sample size

The total number of ED visits in the assigned six centers over a period of 1 month is more than 20,000. We plan to screen around 15,000 patients (75%) with an age range of 18 to 70. Following a traumatic context, 10 to 50% of survivors consult EDs [3, 15, 79, 80]. Considering the most conservative hypothesis (10% of the 15,000 visiting 18–70 year old patients), we estimate that 1500 patients could be included in the study to participate in the cross-sectional part of the study.

The main objective of the cohort study is to identify factors associated with the occurrence of 3-month PTSD. In the literature, incidence of PTSD in various populations and after different types of trauma usually ranges from 30 to 60% [3, 15, 79, 80]. Considering the hypothesis of a 40% incidence of PTSD in the “unexposed” group, the inclusion of 305 patients should allow, with an alpha risk of 0.05, and a power of 80%, to identify factors associated with a relative risk of at least 1.4 [81].

As we anticipate 30% of the subjects may be either lost to follow-up or unwilling to participate at 3 months, we will randomly select a cohort of 400 patients.

### Statistical methods

We use SAS v9.3 software (SAS institute, Cary, NC, USA) for data analysis, and will not impute missing data. A significance level of 5% will be considered for the analysis.

#### Descriptive analysis of the emergency departments and patients participating in the study

Unwillingness of the EDs and/or patients to participate in the study could lead to selection bias. For a critical appraisal of the study findings, we will compare the characteristics of patients included and not included in the cross-sectional study and/or in the cohort study.

Mean and standard deviation (with 95% confidence interval of the mean) will summarize continuous normally distributed variables. Median and interquartile range will summarize continuous non-normally distributed variables. Frequency tables will summarize discrete variables.

There will be a description of characteristics of the two populations studied: total population included in the first cross-sectional phase and the prospective cohort population followed-up at 3 months. Additionally, we will describe and compare characteristics of the subjects who were lost to follow-up to those who completed the follow-up.

#### Primary outcome criteria analysis

To assess the baseline risk of developing PTSD based on the IES-R score (< 12, 12 to 34, > 34) we will calculate the proportion of the subjects at high risk (IES-R score > 34) and moderate-risk (IES-R score 12 to 34) to develop PTSD and their 95% confidence interval.

To analyze the biopsychosocial factors associated with the occurrence of PTSD at 3 months (yes / no) we will employ a univariate model. For statistical testing, we will use the Chi-squared test for qualitative variables, Student’s test for quantitative variables following a normal distribution, Wilcoxon test for quantitative variables following a non-normal distribution, and a Kruskal & Wallis rank test for ordered quantitative variables of the score type. Univariate and multivariate logistic regression modeling will facilitate estimation of the association between the studied factors and the 3-month risk of PTSD by calculating the crude and adjusted odds ratio and their 95% confidence interval.

Among 305 analyzable patients, with a 40% incidence rate of PTSD, we expect 122 patients in the PTSD group. To ensure the convergence and robustness of the statistical model, we will not integrate more than twelve explanatory variables into the multivariate predictive model [82].

#### Secondary outcome criteria analysis

In the cross-sectional study subjects, we will analyze the proportion of patients at moderate risk (IES-R score 12 to 34) and at high risk (IES-R score > 34) of PTSD at inclusion and their 95% confidence interval.

In the cohort study population, we will analyze the proportion of subjects with a diagnosis of PTSD at 3 months with its 95% confidence interval.

To describe results of the questionnaires, we will consider total score of PDEQ, STAI-Y (A & B forms), AUDIT, Fagerström, QIDS-SR16 and SSQ6 evaluated at inclusion. To present the results of the questionnaires at 3 months, we will focus on total score of STAI-Y (A & B forms), AUDIT, Fagerström, QIDS-SR16 and SSQ6 evaluated again after 3 months.

##### Among subjects with 3-month PTSD

To describe PTSD complications and comorbidities, we will consider the proportion of patients with excessive alcohol consumption (AUDIT), the proportion of patients with tobacco addiction and its level (low, medium or high; Fagerström test), the severity of depressive symptoms (QIDS) and the proportion of patients in each of the five categories (from no depression to very severe depression), and the response to item 12 of QIDS-SR16 which will depict proportion of subjects with suicidal ideation.

We will use mean, standard deviation, median, and interquartile range to illustrate the number of days lost from work and the number of suicide attempts over the last 3 months. The proportion of patients with at least one of these events will also be measured.

We will present secondary endpoint results for the total study population and the subgroups according to the level of risk identified at inclusion by the IES–R score (moderate risk = IES-R score 12 to 34; high risk = IES-R score > 34).

## Discussion

### Strengths of the study

Firstly, to the best of our knowledge, this will be the first study to assess prevalence of acute stress and risk of PTSD in diverse trauma survivors visiting the ED due to a recent trauma. Previous studies have focused on a specific trauma such as road traffic accidents or rape victims, etc. Secondly, the prospective design of the study will minimize potential information or recall bias. A number of similar studies have used case-control or retrospective designs, and assessed the subjects after months and in some cases after years, which increases the probability of recall bias. Thirdly, the large sample size of this study will ensure the generalizability of the findings. Small sample size is a very common problem in studies on PTSD; some studies have been conducted on a very low sample size while others studies suffer from huge dropout rates that subsequently. Fourthly, we use a holistic biopsychosocial approach to evaluate PTSD predictive factors, while studies investigating PTSD predictors usually explore a single domain (biological, psychological, social, or demographic). Fifthly, the findings will determine PTSD risk in trauma survivors who have an IES-R score between 12 and 34 on, for whom there is no literature on PTSD vulnerability. Sixthly, the use of PCL-5, a standardized scale for diagnosing PTSD at 3 months, is one of the strengths of this study. Specifically trained staff (psychologist, psychiatrist, or intern in psychiatry) will complete the scale during a telephone interview with the consumer. Seventhly, the results will represent a wide geographical area and its innate diversity through the multicenter nature of the study. Finally, the results will provide carers and healthcare providers with invaluable information for the identification of the population at risk of PTSD and to plan preventive screening and therapeutic procedures.

### Challenges

One challenge that we may probably encounter at the cross-sectional stage is that we will not be able to recruit every patient consulting the EDs. Due to their either unwillingness to participate or failure to meet the inclusion criteria. To address this problem, we will elaborate their respective characteristics in contrast to the patients included.

A second potential challenge will be an unexpected rate of dropout in the cohort stage. In order to address this potential issue, selected subjects will receive reminder letters and/or emails 1 month prior to the telephone interview, and we will send them the self-administered questionnaires with a pre-paid return envelope. In addition, a professional will call or email them 15 days in advance to set the date and time of the interview. In case of “no reply”, three more attempts will be made. Finally, the multicenter nature of the study and recruitment capacity of participating EDs (significantly higher than required) ensure feasibility of recruiting expected number of subjects.

## Data Availability

Not applicable.

## Abbreviations

ASD: Acute Stress Disorder
AUDIT: Alcohol Use Disorder Identification Test
CAPS-IV: Clinician Administered PTSD Scale for DSM-IV
ED: Emergency Department
ePRO: electronic Patient Reported Outcomes
IES-R: Impact of Event Scale-Revised
IPV: Intimate Partner Violence
PCL-5: PTSD Check List for DSM-5
PDEQ: Peri-traumatic Dissociative Experiences Questionnaire
PTSD: Post-Traumatic Stress Disorder
QIDS-SR16: 16-item Quick Inventory of Depressive Symptomatology (Self-Report)
SSQ6: 6-item Social Support Questionnaire
STAI-Y (A + B): State-Trait Anxiety Inventory-Y (form A & B)

## References

[1] Forbes D, Creamer M, Phelps A, Bryant R, McFarlane A, Devilly GJ (2007). Australian guidelines for the treatment of adults with acute stress disorder and post-traumatic stress disorder. Aust N Z J Psychiatry.

[2] DiGangi JA, Gomez D, Mendoza L, Jason LA, Keys CB, Koenen KC (2013). Pretrauma risk factors for posttraumatic stress disorder: a systematic review of the literature. Clin Psychol Rev.

[3] Foa EB, Keane TM, Friedman MJ, Cohen JA. Effective treatments for PTSD, second edition: practice guidelines from the International Society for Traumatic Stress Studies, vol. 673: Guilford Press; 2008.

[4] Association AP. Diagnostic and statistical manual of mental disorders (DSM-5®): American Psychiatric Pub; 2013. 1519 p

[5] NIfCE N. The management of PTSD in adults and children in primary and secondary care. Natl Clin Pract Guidel 2005; 21834189

[6] Reeves RR (2008). Latest strategies in diagnosis and treatment of PTSD. Med Econ.

[7] Ashbaugh AR, Houle-Johnson S, Herbert C, El-Hage W, Brunet A (2016). Psychometric validation of the English and French versions of the posttraumatic stress disorder checklist for DSM-5 (PCL-5). PLoS One.

[8] Blevins CA, Weathers FW, Davis MT, Witte TK, Domino JL (2015). The posttraumatic stress disorder checklist for DSM-5 (PCL-5): development and initial psychometric evaluation. J Trauma Stress.

[9] Palmieri PA, Weathers FW, Difede J, King DW (2007). Confirmatory factor analysis of the PTSD checklist and the clinician-administered PTSD scale in disaster workers exposed to the world trade center ground zero. J Abnorm Psychol.

[10] Husky MM, Lépine J-P, Gasquet I, Kovess-Masfety V (2015). Exposure to traumatic events and posttraumatic stress disorder in France: results from the WMH survey. J Trauma Stress.

[11] Panagioti M, Gooding PA, Tarrier N (2012). A meta-analysis of the association between posttraumatic stress disorder and suicidality: the role of comorbid depression. Compr Psychiatry.

[12] Lee DJ, Liverant GI, Lowmaster SE, Gradus JL, Sloan DM (2014). PTSD and reasons for living: associations with depressive symptoms and alcohol use. Psychiatry Res.

[13] Ahmadi N, Hajsadeghi F, Mirshkarlo HB, Budoff M, Yehuda R, Ebrahimi R (2011). Post-traumatic stress disorder, coronary atherosclerosis, and mortality. Am J Cardiol.

[14] Dedert EA, Calhoun PS, Watkins LL, Sherwood A, Beckham JC (2010). Posttraumatic stress disorder, cardiovascular, and metabolic disease: a review of the evidence. Ann Behav Med.

[15] Bobo WV, Warner CH, Warner CM (2007). The management of post traumatic stress disorder (PTSD) in the primary care setting. South Med J.

[16] Holeva V, Tarrier N, Wells A (2001). Prevalence and predictors of acute stress disorder and PTSD following road traffic accidents: thought control strategies and social support. Behav Ther.

[17] Bryant RA, Harvey AG (1996). Initial posttraumatic stress responses following motor vehicle accidents. J Trauma Stress.

[18] Mayou R, Bryant B, Duthie R (1993). Psychiatric consequences of road traffic accidents. Bmj..

[19] Bryant RA (2005). Predicting posttraumatic stress disorder from acute reactions. J Trauma Dissociation.

[20] Holeva V, Tarrier N (2001). Personality and peritraumatic dissociation in the prediction of PTSD in victims of road traffic accidents. J Psychosom Res.

[21] Forbes D, Wolfgang B, Cooper J, Creamer M, Barton D (2009). Post-traumatic stress disorder--best practice GP guidelines. Aust Fam Physician.

[22] Benedek DM, Friedman MJ, Zatzick D, Ursano RJ (2009). Guideline watch (march 2009): practice guideline for the treatment of patients with acute stress disorder and posttraumatic stress disorder. Focus..

[23] Bomyea J, Risbrough V, Lang AJ (2012). A consideration of select pre-trauma factors as key vulnerabilities in PTSD. Clin Psychol Rev.

[24] Te Brake H, Dückers M, De Vries M, Van Duin D, Rooze M, Spreeuwenberg C (2009). Early psychosocial interventions after disasters, terrorism, and other shocking events: guideline development. Nurs Health Sci.

[25] Bryant RA, Creamer M, O’Donnell M, Silove D, McFarlane AC (2012). The capacity of acute stress disorder to predict posttraumatic psychiatric disorders. J Psychiatr Res.

[26] Bui Eric, Brunet Alain, Allenou Charlotte, Camassel Cécile, Raynaud Jean-Philippe, Claudet Isabelle, Fries Frédéric, Cahuzac Jean-Philippe, Grandjean Hélène, Schmitt Laurent, Birmes Philippe (2010). Peritraumatic reactions and posttraumatic stress symptoms in school-aged children victims of road traffic accident. General Hospital Psychiatry.

[27] Cukor J, Spitalnick J, Difede J, Rizzo A, Rothbaum BO (2009). Emerging treatments for PTSD. Clin Psychol Rev.

[28] Ehring T, Ehlers A, Cleare AJ, Glucksman E (2008). Do acute psychological and psychobiological responses to trauma predict subsequent symptom severities of PTSD and depression?. Psychiatry Res.

[29] Gavranidou M, Rosner R (2003). The weaker sex? Gender and post-traumatic stress disorder. Depress Anxiety.

[30] Gelder M, Harrison P, Cowen P. Shorter Oxford textbook of psychiatry Oxford University press. N Y. 2006;137.

[31] Heron-Delaney M, Kenardy J, Charlton E, Matsuoka Y (2013). A systematic review of predictors of posttraumatic stress disorder (PTSD) for adult road traffic crash survivors. Injury..

[32] Walsh K, Nugent NR, Kotte A, Amstadter AB, Wang S, Guille C (2013). Cortisol at the emergency room rape visit as a predictor of PTSD and depression symptoms over time. Psychoneuroendocrinology..

[33] Scott-Tilley D, Tilton A, Sandel M (2010). Biologic correlates to the development of post-traumatic stress disorder in female victims of intimate partner violence: implications for practice. Perspect Psychiatr Care.

[34] Russo J, Katon W, Zatzick D (2013). The development of a population-based automated screening procedure for PTSD in acutely injured hospitalized trauma survivors. Gen Hosp Psychiatry.

[35] Miller AB, Schaefer KE, Renshaw KD, Blais RK (2013). PTSD and marital satisfaction in military service members: examining the simultaneous roles of childhood sexual abuse and combat exposure. Child Abuse Negl.

[36] Kuhn E, Blanchard EB, Fuse T, Hickling EJ, Broderick J (2006). Heart rate of motor vehicle accident survivors in the emergency department, peritraumatic psychological reactions, ASD, and PTSD severity: a 6-month prospective study. J Trauma Stress Off Publ Int Soc Trauma Stress Stud..

[37] Naeem F, Ayub M, Masood K, Gul H, Khalid M, Farrukh A (2011). Prevalence and psychosocial risk factors of PTSD: 18 months after Kashmir earthquake in Pakistan. J Affect Disord.

[38] Hopwood M, Howard S (2003). Post-traumatic stress disorder: a brief overview. Aust Fam Physician.

[39] Mealer M, Burnham EL, Goode CJ, Rothbaum B, Moss M (2009). The prevalence and impact of post traumatic stress disorder and burnout syndrome in nurses. Depress Anxiety..

[40] Michopoulos V, Norrholm SD, Jovanovic T (2015). Diagnostic biomarkers for posttraumatic stress disorder: promising horizons from translational neuroscience research. Biol Psychiatry.

[41] Reich J, Lyons M, Cai B (1996). Familial vulnerability factors to post-traumatic stress disorder in male military veterans. Acta Psychiatr Scand.

[42] Blanchard EB, Hickling EJ, Taylor AE, Loos WR, Forneris CA, Jaccard J (1996). Who develops PTSD from motor vehicle accidents?. Behav Res Ther.

[43] Landolt MA, Vollrath M, Timm K, Gnehm HE, Sennhauser FH (2005). Predicting posttraumatic stress symptoms in children after road traffic accidents. J Am Acad Child Adolesc Psychiatry.

[44] Haagsma JA, Ringburg AN, van Lieshout EM, van Beeck EF, Patka P, Schipper IB (2012). Prevalence rate, predictors and long-term course of probable posttraumatic stress disorder after major trauma: a prospective cohort study. BMC Psychiatry.

[45] Creamer M, O’Donnell ML, Pattison P (2004). The relationship between acute stress disorder and posttraumatic stress disorder in severely injured trauma survivors. Behav Res Ther.

[46] Vranceanu A-M, Hobfoll SE, Johnson RJ (2007). Child multi-type maltreatment and associated depression and PTSD symptoms: the role of social support and stress. Child Abuse Negl.

[47] Haden SC, Scarpa A, Jones RT, Ollendick TH (2007). Posttraumatic stress disorder symptoms and injury: the moderating role of perceived social support and coping for young adults. Personal Individ Differ.

[48] MacGregor AJ, Corson KS, Larson GE, Shaffer RA, Dougherty AL, Galarneau MR (2009). Injury-specific predictors of posttraumatic stress disorder. Injury..

[49] Saigh PA (1991). The development of posttraumatic stress disorder following four different types of traumatization. Behav Res Ther.

[50] Delahanty DL, Raimonde AJ, Spoonster E (2000). Initial posttraumatic urinary cortisol levels predict subsequent PTSD symptoms in motor vehicle accident victims. Biol Psychiatry.

[51] Vaiva G, Ducrocq F, Jezequel K, Averland B, Lestavel P, Brunet A (2003). Immediate treatment with propranolol decreases posttraumatic stress disorder two months after trauma. Biol Psychiatry.

[52] McFarlane AC, Barton CA, Yehuda R, Wittert G (2011). Cortisol response to acute trauma and risk of posttraumatic stress disorder. Psychoneuroendocrinology..

[53] El-Jawahri AR, Vandusen HB, Traeger LN, Fishbein JN, Keenan T, Gallagher ER (2016). Quality of life and mood predict posttraumatic stress disorder after hematopoietic stem cell transplantation. Cancer..

[54] Delahanty DL, Raimonde AJ, Spoonster E, Cullado M (2003). Injury severity, prior trauma history, urinary cortisol levels, and acute PTSD in motor vehicle accident victims. J Anxiety Disord.

[55] Zatzick DF, Russo J, Pitman RK, Rivara F, Jurkovich G, Roy-Byrne P (2005). Reevaluating the association between emergency department heart rate and the development of posttraumatic stress disorder: a public health approach. Biol Psychiatry.

[56] Morina N, Ehring T, Priebe S (2013). Diagnostic utility of the impact of event scale–revised in two samples of survivors of war. PLoS One.

[57] Rash CJ, Coffey SF, Baschnagel JS, Drobes DJ, Saladin ME (2008). Psychometric properties of the IES-R in traumatized substance dependent individuals with and without PTSD. Addict Behav.

[58] Bienvenu OJ, Williams JB, Yang A, Hopkins RO, Needham DM (2013). Posttraumatic stress disorder in survivors of acute lung injury: evaluating the impact of event scale-revised. Chest..

[59] Adkins JW, Weathers FW, McDevitt-Murphy M, Daniels JB (2008). Psychometric properties of seven self-report measures of posttraumatic stress disorder in college students with mixed civilian trauma exposure. J Anxiety Disord..

[60] Jeantieu M, Gaillat F, Antonini F, Azoulay E, Martin C, Thomas P (2014). Postoperative pain and subsequent PTSD-related symptoms in patients undergoing lung resection for suspected cancer. J Thorac Oncol.

[61] Asukai N, Kato H, Kawamura N, Kim Y, Yamamoto K, Kishimoto J (2002). Reliabiligy and validity of the Japanese-language version of the impact of event scale-revised (Ies-RJ): four studies of different traumatic events. J Nerv Ment Dis.

[62] Creamer M, Bell R, Failla S (2003). Psychometric properties of the impact of event scale—revised. Behav Res Ther.

[63] de Santé HA (2007). Affections psychiatriques de longue durée. Troubles anxieux graves. St-Denis Plaine HAS.

[64] Elhai JD, Gray MJ, Kashdan TB, Franklin CL (2005). Which instruments are most commonly used to assess traumatic event exposure and posttraumatic effects?: a survey of traumatic stress professionals. J Trauma Stress Off Publ Int Soc Trauma Stress Stud.

[65] Brunet A, St-Hilaire A, Jehel L, King S (2003). Validation of a French version of the impact of event scale-revised. Can J Psychiatr.

[66] Wortmann JH, Jordan AH, Weathers FW, Resick PA, Dondanville KA, Hall-Clark B (2016). Psychometric analysis of the PTSD Checklist-5 (PCL-5) among treatment-seeking military service members. Psychol Assess.

[67] Bovin MJ, Marx BP, Weathers FW, Gallagher MW, Rodriguez P, Schnurr PP (2016). Psychometric properties of the PTSD checklist for diagnostic and statistical manual of mental disorders–fifth edition (PCL-5) in veterans. Psychol Assess.

[68] Spielberger CD, Gorsuch RL, Lushene R, Vagg PR, Jacobs GA. Manual for the State-trait anxiety inventory (form Y self-evaluation questionnaire) consulting psychologists press: Palo Alto. CA. 1983.

[69] Gauthier J, Bouchard S (1993). French-Canadian Adaptation Of A Revised Form Of The State-Trait Anxiety Inventory By Spielberger. Can J Behav Sci-Rev Can Sci Comport.

[70] Birmes P, Brunet A, Benoit M, Defer S, Hatton L, Sztulman H (2005). Validation of the Peritraumatic dissociative experiences questionnaire self-report version in two samples of French-speaking individuals exposed to trauma. Eur Psychiatry.

[71] Sarason IG, Levine HM, Basham RB, Sarason BR (1983). Assessing social support: the social support questionnaire. J Pers Soc Psychol.

[72] Bruchon-Schweitzer M, Rascle N, Cousson-Gélie F, Bidan-Fortier C, Sifakis Y, Constant A (2003). Le questionnaire de soutien social de Sarason (SSQ6). Une adaptation française Psychol Fr.

[73] Gache P, Michaud P, Landry U, Accietto C, Arfaoui S, Wenger O (2005). The alcohol use disorders identification test (AUDIT) as a screening tool for excessive drinking in primary care: reliability and validity of a French version. Alcohol Clin Exp Res.

[74] Heatherton TF, Kozlowski LT, Frecker RC, Fagerstrom K-O (1991). The Fagerström test for nicotine dependence: a revision of the Fagerstrom tolerance questionnaire. Br J Addict.

[75] Aubin PH-J, Gillet C, Rigaud A (2015). Mésusage de l’alcool dépistage, diagnostic et traitement. Alcoologie Addictologie.

[76] Surís A, Holder N, Holliday R, Clem M (2016). Psychometric validation of the 16 item quick inventory of depressive symptomatology self-report version (QIDS-SR16) in military veterans with PTSD. J Affect Disord.

[77] Rush AJ, Trivedi MH, Ibrahim HM, Carmody TJ, Arnow B, Klein DN (2003). The 16-item quick inventory of depressive symptomatology (QIDS), clinician rating (QIDS-C), and self-report (QIDS-SR): a psychometric evaluation in patients with chronic major depression. Biol Psychiatry.

[78] Trivedi MH, Rush AJ, Ibrahim HM, Carmody TJ, Biggs MM, Suppes T (2004). The inventory of depressive symptomatology, clinician rating (IDS-C) and self-report (IDS-SR), and the quick inventory of depressive symptomatology, clinician rating (QIDS-C) and self-report (QIDS-SR) in public sector patients with mood disorders: a psychometric evaluation. Psychol Med.

[79] Tsujiuchi T, Yamaguchi M, Masuda K, Tsuchida M, Inomata T, Kumano H (2016). High prevalence of post-traumatic stress symptoms in relation to social factors in affected population one year after the Fukushima nuclear disaster. PLoS One.

[80] Skogstad L, Tøien K, Hem E, Ranhoff AH, Sandvik L, Ekeberg Ø (2014). Psychological distress after physical injury: a one-year follow-up study of conscious hospitalised patients. Injury..

[81] Schlesselman JJ (1974). Sample size requirements in cohort and case-control studies of disease. Am J Epidemiol.

[82] Peduzzi P, Concato J, Feinstein AR, Holford TR (1995). Importance of events per independent variable in proportional hazards regression analysis II. Accuracy and precision of regression estimates. J Clin Epidemiol.

